# Persistence on Oral HIV Pre‐Exposure Prophylaxis Among Key Populations at a Centre in Buenos Aires: A Survival Analysis of a Real‐World Cohort

**DOI:** 10.1002/jia2.70175

**Published:** 2026-07-25

**Authors:** Julian L. Garcia, Diego Salusso, Gissella Mernies, María M. Sandoval, Carla Petriglieri, Felipe Bilbao, Daniela Parera, Carolina Perez, Adriana Duran, Ines Aristegui, Maria Ines Figueroa, Carina Cesar

**Affiliations:** ^1^ Research Department Fundación Huésped Buenos Aires Argentina; ^2^ Coordination of Sexual Health HIV and Sexually Transmitted Infections Ministry of Health Buenos Aires Argentina

**Keywords:** HIV, Latin America, MSM, pre‐exposure prophylaxis, sexual and gender minorities, transgender person

## Abstract

**Introduction:**

Although HIV pre‐exposure prophylaxis (PrEP) has been rapidly scaled up in Argentina to reach key populations with high HIV burden, evidence on real‐world persistence is lacking. Understanding who remains engaged in care, and why others discontinue, is essential to ensure the effectiveness of PrEP. This study evaluated oral PrEP persistence and identified demographic and behavioural factors associated with discontinuation in a cohort of individuals initiating PrEP in Buenos Aires.

**Methods:**

We performed a retrospective cohort study including all users initiating daily or event‐driven oral PrEP at our centre between September 2021 and July 2025. The cohort consisted of transgender women (TGW), men who have sex with men (MSM) and cisgender women engaged in sex work (cSW), in line with national PrEP guidelines. Persistence on PrEP was defined as the time to first discontinuation (TFD), either self‐reported or estimated as the date when the last dispensed PrEP pill would have been exhausted if taken daily. Categories for discontinuation included participant‐reported causes and loss to follow‐up (LTFU). TFD was evaluated using survival analysis. Cox proportional hazards models were fitted to examine associations between TFD and sociodemographic and behavioural factors.

**Results:**

Overall, 970 individuals initiated PrEP: 69% MSM, 24% TGW and 6% cSW. Median age was 31 years (IQR 26–36). During follow‐up, 447 participants (46%) discontinued PrEP. Persistence declined steadily over time: 91% at 1 month, 81% at 6 months, 72% at 12 months, 58% at 24 months and 43% at 36 months. Main categories for discontinuation were LTFU (44%), user decision or perceived low risk (46% combined) and adverse events (10%). In the multivariable model, younger age, identifying as TGW or cSW, cocaine use in the last month before baseline and the absence of basal/follow‐up syphilis or other bacterial sexually transmitted infections were associated with PrEP discontinuation.

**Conclusions:**

Nearly half of the cohort discontinued PrEP over 3 years, with differences across key populations. These findings highlight the need for differentiated community‐tailored PrEP delivery models that address structural and behavioural barriers to improve long‐term PrEP engagement among key populations in Argentina and wider Latin America.

**Abbreviations**
AEs: adverse events
CABA: Buenos Aires City
cSW: cisgender women engaged in sex work
FTC/TDF: emtricitabine and tenofovir disoproxil difumarate
HIV: human immunodeficiency virus
LTFU: lost to follow‐up
MSM: men who have sex with men
PEP: post‐exposure prophylaxis
PLWH: people living with HIV
PrEP: pre‐exposure prophylaxis
STI(s): sexually transmitted infection(s)
SW: sexual worker
TFD: time to first discontinuation
TGW: transgender women

## 1 Introduction

The global expansion of HIV pre‐exposure prophylaxis (PrEP) is critical to reaching UNAIDS targets and ending the AIDS epidemic [1]. In Argentina, 140,000 people are living with HIV (PLWH), with 6900 new diagnoses each year disproportionately affecting key populations [2]. Estimated HIV prevalence is 34% among transgender women (TGW), 12%–15% among men who have sex with men (MSM) and 2%–5% among sex workers (SWs) [3]. Although robust HIV incidence estimates are lacking in Argentina, studies from Latin America, North America and other regions consistently show high incidence among MSM and TGW, ranging from 2 to 9 per 100 person‐years in trials and observational cohorts [4, 5, 6]. These data underscore the substantial risk faced by these groups and the need for local evidence on PrEP engagement and persistence to inform prevention strategies.

Oral PrEP with emtricitabine/tenofovir disoproxil fumarate (FTC/TDF) is a highly effective strategy for preventing HIV acquisition [7]. Its efficacy has been demonstrated across diverse populations and settings, leading to its approval by the U.S Food and Drug Administration (FDA) in 2012 [8] and recommendation by the World Health Organization (WHO) in 2015 [9]. Several countries have reported declines in HIV incidence following the rollout of national PrEP programmes targeting key populations [10]. However, real‐world impact depends not only on access but also on models of service delivery. Integration into comprehensive, community‐tailored health services improves access among those most vulnerable [11, 12].

Persistence in PrEP programmes, defined as continued engagement with PrEP care over time, is essential for real‐world effectiveness. However, maintaining PrEP use remains challenging, especially in low‐ and middle‐income countries, where structural and behavioural barriers can undermine long‐term uptake and impact [13]. Observational studies in Latin America and other regions have shown that younger age, low educational attainment, unstable housing and stigma are consistently associated with lower persistence [14]. TGW, in particular, face high discontinuation rates linked to discrimination and limited access to gender‐affirming care [15, 16, 17, 18].

In Argentina, oral PrEP was introduced as a national initiative in September 2021, initially targeting MSM, TGW and SW [19]. By the end of 2024, 5376 individuals had enrolled [Ministry of Health, unpublished]; however, no published data are available on user characteristics or persistence outcomes, representing a critical knowledge gap for both Argentina and the region. Such evidence is essential to inform differentiated service delivery models, strengthen retention and optimize PrEP preventive potential.

This study aims to evaluate persistence on daily and event‐driven oral PrEP and to identify demographic and behavioural characteristics associated with discontinuation in a real‐world cohort of individuals initiating oral PrEP at a centre in Buenos Aires, Argentina.

## 2 Methods

### 2.1 Study Design and Setting

The national PrEP programme was launched in September 2021. This retrospective cohort study included individuals enrolled in a PrEP service delivery programme at Fundación Huésped, a non‐governmental organization in Buenos Aires, Argentina, providing free HIV prevention and care services to individuals at high risk of HIV acquisition. All individuals initiating PrEP at the centre between September 2021 and July 2025 were eligible for inclusion. Data were collected as part of routine clinical care, and follow‐up was conducted according to national PrEP guidelines [19].

### 2.2 Eligibility Criteria and Recruitment

PrEP was offered to individuals without HIV identified as being at high risk for HIV acquisition. Eligibility criteria included: (1) MSM or TGW reporting condomless anal sex, a bacterial sexually transmitted infection (STI) within the previous 6 months or more than one use of post‐exposure prophylaxis (PEP); (2) individuals in serodifferent couples engaging in condomless sex when the partner with HIV did not have a sustained viral suppression; or (3) cisgender women engaged in sex work (cSW) or any individuals engaged in sex work.

PrEP was offered to individuals seeking HIV testing or PEP at our centre, and through peer referrals. Provider‐driven initiation referred to cases in which PrEP was primarily recommended by the healthcare provider, whereas user‐driven initiation referred to cases in which participants proactively requested PrEP. In all cases, initiation required a medical prescription. Eligible participants received same‐day oral PrEP—fixed dose tenofovir disoproxil fumarate 300 mg/emtricitabine 200 mg—either as a daily or event‐driven regimen. Daily PrEP was offered to TGW using gender‐affirming hormone therapy and cisgender women engaged in sex work. MSM and TGW not using gender‐affirming hormone therapy could choose daily or event‐driven PrEP after counselling.

### 2.3 Clinical Assessment and Data Collection

At baseline and follow‐up visits, participants completed a structured self‐administered questionnaire collecting sociodemographic and behavioural information, including sexual practices, condom and substance use, STI history, and self‐reported PrEP adherence, assessed by missed PrEP pills in the previous month. A safety visit was conducted 1 month after PrEP initiation to evaluate early tolerability and exclude acute HIV infection. Follow‐up visits were scheduled every 3 months and included questionnaire‐based and physician‐led assessment of adherence, concomitant medication, adverse events (AEs), incident STIs and HIV seroconversion. Additional visits were arranged as clinically indicated. Participants underwent HIV, syphilis and viral hepatitis (A, B, C) serology, and renal and liver function tests. *Neisseria gonorrhoeae* and *Chlamydia trachomatis* testing was performed in symptomatic participants at baseline and repeated every 6 months. HIV testing was conducted at every visit. Reactive HIV tests were confirmed by plasma HIV‐RNA testing. Data were captured and securely stored in a REDCap electronic platform [20].

Appointment reminders were routinely sent 1 day before scheduled visits. For participants missing visits, up to three contact attempts were made via phone or email before classifying them as loss to follow‐up (LTFU).

### 2.4 Operational Definitions

Persistence on PrEP was defined as the time to first discontinuation (TFD). Discontinuation was classified hierarchically as: (1) participant‐reported reasons (user decision, perceived low risk of HIV acquisition and AEs); (2) HIV acquisition; and (3) LTFU, defined as no visit occurred within 90 days of the scheduled appointment plus unsuccessful contact attempts. For LTFU, TFD was estimated as the date when the last PrEP pill dispensed would have been exhausted if taken daily; the same operational definition was applied to event‐driven PrEP users. In a sensitivity analysis, participants classified as LTFU in the primary analysis were censored at the date of their last recorded visit.

Syphilis was defined as clinical or serological diagnosis at baseline (±30 days) or during follow‐up (i.e. incident cases). Other bacterial STIs were defined as clinical events compatible with urethritis, proctitis, genital or anal ulcer; or laboratory‐confirmed *Neisseria gonorrhoeae* or *Chlamydia trachomatis* infection at baseline (±30 days) or during follow‐up (i.e. incident cases). Syphilis and other bacterial STIs were also defined based on recorded treatment.

Sociodemographic characteristics included key population group (MSM, TGW, cSW), age, country of birth, residence (Buenos Aires city [CABA] vs. outside CABA), healthcare coverage (private/public) and educational attainment (complete high‐school or higher vs. incomplete high‐school or lower). Place of residence (CABA vs. outside CABA) was included as a contextual variable because, in our setting, seeking PrEP care outside one's residential area may reflect structural barriers such as stigma, confidentiality concerns or discrimination in local services, while longer travel distances may hinder follow‐up continuity and retention. Behavioural characteristics included drug use in the month before baseline (any substance; stimulants [cocaine, cocaine paste/base], chemsex‐associated drugs [poppers, sildenafil/tadalafil, GHB/hydroxybutyrate, ketamine, mephedrone and ecstasy/MDMA/amphetamines], hallucinogens [LSD], opioids [heroin] and marijuana), cocaine use before discontinuation (reported at the last visit), lifetime sexual work (ever exchanging sex for money, gifts, protection or help), history of STIs—including syphilis, hepatitis B, hepatitis C, gonorrhoea or chlamydia—, number of sexual partners in the month before baseline and PrEP initiation (user vs. provider‐driven).

### 2.5 Statistical Analysis

Baseline categorical variables were summarized as frequencies and percentages (excluding missing data), and continuous variables as medians and interquartile ranges (IQRs). Comparisons across key populations and between participants who discontinued versus remained on PrEP were performed using the Kruskal–Wallis test for continuous or ordinal variables, and chi‐square or Fisher's exact test for categorical variables, as appropriate.

Persistence on PrEP was analysed using survival analysis methods. Kaplan−Meier curves were used to assess the TFD for the total study population and stratified by relevant covariates. Bivariate analysis was assessed by a log‐rank test.

Factors associated with PrEP discontinuation were assessed using Cox proportional hazards models, with TFD as the outcome. Covariates included age, key population group, residence, educational attainment, lifetime sex work, drug use before discontinuation, user‐ versus provider‐driven PrEP initiation, and syphilis and/or other bacterial STIs at baseline and/or during follow‐up. Hazard ratios (HRs) with 95% confidence intervals (CIs) were reported. Effect modification by key population was assessed through interaction terms with covariates associated with discontinuation.

### 2.6 Ethical Considerations

This study involved secondary analysis of anonymized, routinely collected clinical data. In line with public health protocols, participants provided verbal clinical consent at the time of PrEP initiation. The use of de‐identified data for research purposes was approved by the Fundación Huésped Institutional Ethics Committee (28‐May‐2025), in compliance with the Declaration of Helsinki and CIOMS guidelines. Written informed consent was not required as per national regulations for minimal‐risk observational studies (Resolution 1480/2011 of the Argentine Ministry of Health).

## 3 Results

A total of 970 individuals initiated oral PrEP; 3% received an event‐driven prescription and 97% a daily prescription. Overall, 69% were MSM (*n* = 673), 24% TGW (*n* = 236) and 6% cSW (*n* = 61). Baseline characteristics are summarized in Table 1. The median age was 31 years (IQR: 26–36); higher in cSW and lower for TGW (*p* < 0.001). Overall, 84% of participants had completed high‐school and 53% had public healthcare coverage. MSM had higher educational attainment and greater access to private healthcare (96% and 63%) compared to TGW (56% and 15%) and cSW (54% and 5%) (*p* < 0.001). Most participants (77%) lived in CABA, with higher proportions among TGW (83%) and MSM (78%) than cSW (54%, *p* < 0.001). A total of 31% were born outside Argentina, most commonly in Venezuela, Perú, Brazil, Paraguay and Colombia; this proportion was higher among MSM (34%, *p*: 0.043).

**TABLE 1:** Baseline characteristics of study participants.

Characteristic	Overall *N* = 970	MSM *N* = 673	TGW *N* = 236	cSW *N* = 61	*p*‐value^c^
Age (years)^a^	31 (26, 36)	31 (27, 36)	29 (24, 34)	37 (29, 44)	< 0.001
Country of birth^b^					0.043
Argentina	666 (69%)	446 (66%)	177 (75%)	43 (70%)	
Other	304 (31%)	227 (34%)	59 (25%)	18 (30%)	
Place of residence^b^					< 0.001
CABA	751 (77%)	522 (78%)	196 (83%)	33 (54%)	
Outside CABA	219 (23%)	151 (22%)	40 (17%)	28 (46%)	
Healthcare coverage^b^					< 0.001
Private	459 (47%)	421 (63%)	35 (15%)	3 (5%)	
Public	511 (53%)	252 (37%)	201 (85%)	58 (95%)	
Education attainment^b^					< 0.001
Complete high‐school or higher	810 (84%)	646 (96%)	131 (56%)	33 (54%)	
Incomplete high‐school or lower	160 (16%)	27 (4.0%)	105 (44%)	28 (46%)	
Drug use in the last month before baseline^b^	443 (52%)	320 (54%)	99 (50%)	24 (40%)	0.100
Missing data	116	78	37	1	
Marijuana use in the last month before baseline^b^	385 (45%)	268 (45%)	93 (47%)	24 (40%)	0.700
Missing data	116	78	37	1	
Chemsex‐associated drugs use in the last month before baseline^b^	219 (26%)	191 (32%)	21 (11%)	7 (12%)	< 0.001
Missing data	116	78	37	1	
Cocaine use in the last month before baseline^b^	136 (16%)	49 (8.2%)	68 (34%)	19 (32%)	< 0.001
Missing data	116	78	37	1	
LSD use in the last month before baseline^b^	31 (3.6%)	26 (4.4%)	3 (1.5%)	2 (3.3%)	0.200
Missing data	116	78	37	1	
History of STIs^b^	476 (49%)	324 (48%)	139 (59%)	13 (22%)	< 0.001
Missing data	1	0	0	1	
Syphilis at baseline^b^	101 (10%)	57 (8.5%)	42 (18%)	2 (3%)	< 0.001
Other bacterial STIs at baseline^b^	36 (4%)	26 (4%)	6 (2%)	4 (7%)	0.300
Number of sexual partners in the last month before baseline^a^	5 (3, 12)	4 (2, 8)	15 (5, 30)	12 (7, 40)	< 0.001
Missing data	197	148	45	4	
Lifetime sexual work^b^	376 (39%)	110 (16%)	205 (87%)	61 (100%)	< 0.001
Missing data	2	1	1	0	
PrEP prescription^b^					0.120
Event‐driven	28 (3%)	24 (4%)	4 (2%)	0 (0%)	
Daily	850 (97%)	581 (96%)	208 (98%)	61 (100%)	
Missing data	92	68	24	0	

Abbreviations: CABA, Buenos Aires City; cSW, cisgender women engaged in sex work; MSM, men who have sex with men; STIs, sexually transmitted infections; TGW, transgender women.

^a^ Values are median (Q1, Q3) for continuous variables.

^b^ Values are *n* (%) for categorical variables.

^c^ Pearson's chi‐squared test; Kruskal−Wallis rank sum test.

### 3.1 Substance Use and Sexual Health Indicators

At baseline, 52% reported substance use in the past month, most commonly marijuana (45% of participants), followed by chemsex‐associated drugs (26%), cocaine (16%) and LSD (4%). Cocaine use was more frequent among TGW (34%) and cSW (32%) (*p* < 0.001), whereas MSM more often reported chemsex‐associated drug use (32%) (*p* < 0.001).

Overall, 49% reported a history of STIs, with higher prevalence among TGW (59%) and MSM (48%) (*p* < 0.001). The median number of sexual partners in the past month was 5 (IQR: 3–12), higher among TGW (median 15) and cSW (median 12) (*p* < 0.001). Lifetime sex work was reported by 38% of participants, higher among TGW (87%) and cSW (100%) (*p* < 0.001).

Baseline syphilis prevalence was 10%, higher among TGW (18%) compared to MSM (8%) and cSW (3%) (*p* < 0.001). The prevalence of other bacterial STIs was 4%, with no significant differences across key population groups (*p*: 0.300). At baseline and during follow‐up, 33% of the participants presented syphilis, with higher frequency among MSM (34%) and TGW (35%) comparing to cSW (11%) (*p*: 0.001); other bacterial STIs were observed in 23%, with higher frequency among MSM (28%) comparing to TGW (12%) and cSW (8%) (*p* < 0.001).

Compared with participants who remained on PrEP, those who discontinued were younger (median age 29 years [IQR: 24–35] vs. 31 [IQR: 27–37], *p*< 0.001), more frequently cSW or TGW (12% and 35%, respectively; *p*< 0.001), had lower educational attainment and had more often public healthcare coverage (*p*< 0.001) (Table 2). Cocaine use and lifetime sex work were more common among those who discontinued (22% vs. 11% and 52% vs. 27%, respectively; *p*< 0.001); whereas chemsex‐associated drug use, syphilis and other bacterial STIs were more frequent among participants who remained on PrEP (32% vs. 19%, 38% vs. 27% and 29% vs. 15%, respectively; all *p*< 0.001).

**TABLE 2:** Baseline characteristics of study participants by discontinuation status.

Characteristic	Overall *N* = 970	Not discontinued *N* = 523	Discontinued *N* = 447	*p*‐value^c^
Key population^b^				< 0.001
MSM	673 (69%)	435 (83%)	238 (53%)	
TGW	236 (24%)	79 (15%)	157 (35%)	
cSW	61 (6%)	9 (2%)	52 (12%)	
Age (years)^a^	31 (26, 36)	31 (27, 37)	29 (24, 35)	< 0.001
Country of birth^b^				0.700
Argentina	666 (69%)	362 (69%)	304 (68%)	
Other	304 (31%)	161 (31%)	143 (32%)	
Place of residence^b^				0.300
CABA	751 (77%)	412 (79%)	339 (76%)	
Outside CABA	219 (23%)	111 (21%)	108 (24%)	
Healthcare coverage^b^				< 0.001
Private	459 (47%)	305 (58%)	154 (34%)	
Public	511 (53%)	218 (42%)	293 (66%)	
Educational attainment^b^				
Complete high‐school or higher	810 (84%)	477 (91%)	333 (74%)	< 0.001
Incomplete high‐school or lower	160 (16%)	46 (8.8%)	114 (26%)	
Drug use in the last month before baseline^b^	443 (52%)	272 (52%)	232 (52%)	>0.900
Missing data	116	74	42	
Marijuana use in the last month before baseline^b^	385 (45%)	207 (46%)	178 (44%)	0.500
Missing data	116	74	42	
Chemsex‐associated drugs use in the last month before baseline^b^	219 (26%)	144 (32%)	75 (19%)	< 0.001
Missing data	116	74	42	
Cocaine use in the last month before baseline^b^	136 (16%)	48 (11%)	88 (22%)	< 0.001
Missing data	116	74	42	
LSD use in the last month before baseline^b^	31 (4%)	15 (3%)	16 (4%)	0.600
Missing data	116	74	42	
History of STIs^b^	476 (49%)	256 (49%)	220 (49%)	>0.900
Missing data	1	0	1	
Syphilis at baseline/incident^b^	320 (33%)	198 (38%)	122 (27%)	< 0.001
Other bacterial STIs at baseline or incident^b^	223 (23%)	154 (29%)	69 (15%)	< 0.001
Number of sexual partners in the last month before baseline^a^	5 (3, 12)	5 (3, 10)	6 (2, 15)	0.092
Missing data	197	116	81	
Lifetime sexual work^b^	376 (39%)	143 (27%)	233 (52%)	< 0.001
Missing data	2	2	0	

Abbreviations: CABA, Buenos Aires City; cSW, cisgender women engaged in sex work; MSM, men who have sex with men; STIs, sexually transmitted infections; TGW, transgender women.

^a^ Values are median (Q1, Q3) for continuous variables.

^b^ Values are *n* (%) for categorical variables.

^c^ Pearson's chi‐squared test; Kruskal−Wallis rank sum test.

### 3.2 PrEP Persistence and Discontinuation

PrEP initiation was user‐driven in 62% of cases and provider‐driven in 38%, with the latter more frequent among cSW (77%) and TGW (56%) than MSM (28%) (*p <*0.001).

**FIGURE 1:**
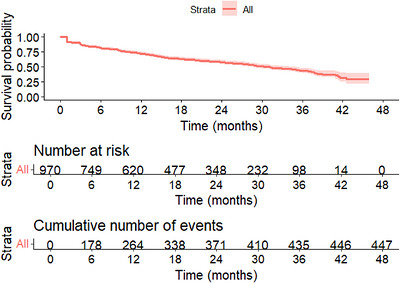
Kaplan–Meier curves of time to first PrEP discontinuation. Kaplan–Meier survival estimates show the probability of remaining on PrEP over time. The lower panels display the number of participants at risk and the cumulative number of discontinuation events at each time point. Time is shown in months.

**FIGURE 2:**
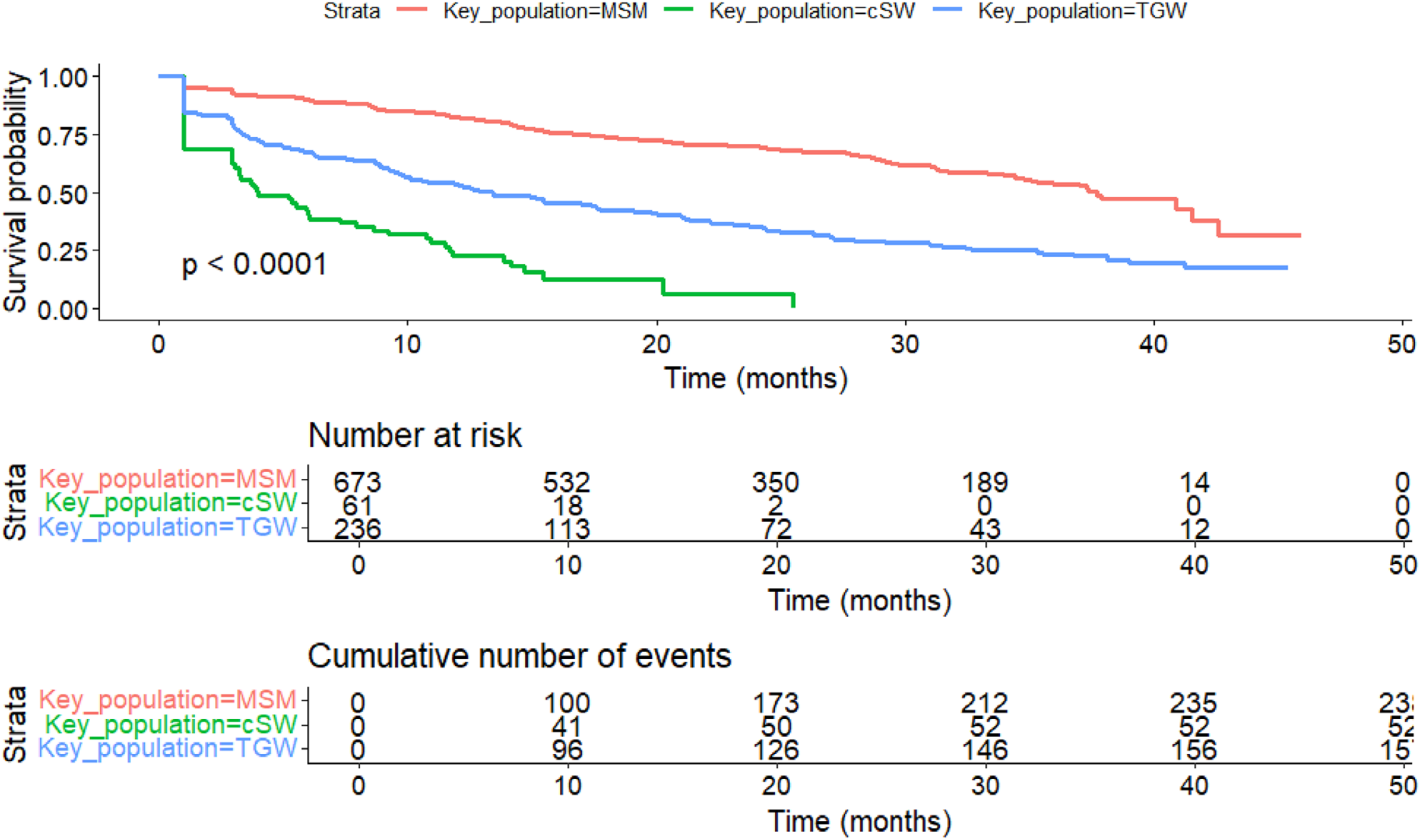
Kaplan−Meier curves of PrEP persistence by key population group. Kaplan–Meier survival estimates show the probability of remaining on PrEP over time among cisgender sex workers (cSW), men who have sex with men (MSM) and transgender women (TGW). Persistence was significantly different across groups (log‐rank test, *p* < 0.0001). The lower panels display the number of participants at risk and the cumulative number of discontinuation events at each time point. Time is shown in months.

Among all users, 46% discontinued at least once over a median follow‐up of 18 months (IQR: 8–29). Persistence declined steadily: 91% remained on PrEP at 1 month (95% CI 89%–93%), 81% at 6 months (79%–84%), 72% at 12 months (69%–75%) and 43% at 36 months (39%–48%) (Figure 1). Persistence was significantly different across key populations (*p* < 0.0001) (Figure 2). The most frequent discontinuation categories were LTFU (44%), user decision or perceived low risk of HIV acquisition (32%) and AEs (10%), mainly mild gastrointestinal symptoms (72%), rash (14%), headache and insomnia (9%), and others (6%). HIV acquisition accounted for 4% of discontinuations.

### 3.3 Factors Associated With PrEP Discontinuation

In bivariate analysis, PrEP discontinuation was significantly associated with identifying as cSW (HR = 6.7, 95% CI: 4.91−9.13) or TGW (HR = 2.63, 95% CI: 2.15−3.23, *p* < 0.001), younger age (HR = 1.03, 95% CI: 1.01−1.04, *p* < 0.001), incomplete high‐school or lower (HR = 2.25, 95% CI: 1.82−2.79, *p* < 0.001), lifetime sex work (HR = 2.37, 95% CI: 1.97−2.86, *p* < 0.001), cocaine use in the last month before baseline (HR = 2.13, 95% CI: 1.68−2.69, *p* < 0.001), chemsex‐associated drugs use in the last month before baseline (HR = 0.59, 95% CI: 0.46−0.76, *p* < 0.001), absence of basal or follow‐up syphilis (HR = 1.84, 95% CI: 1.49−2.27, *p* < 0.001), absence of basal or follow‐up other bacterial STIs (HR = 2.32, 95% CI: 1.80−3.01, *p* < 0.001) and provider‐driven PrEP (HR = 1.60, 95% CI: 1.33−1.93, *p* < 0.001). Effect modification by key population was identified for three variables, using MSM as the reference group. The association between younger age and discontinuation was attenuated among cSW (interaction HR = 0.96, *p* = 0.007). Similarly, the association of lifetime sex work among TGW (interaction HR = 0.41, *p* = 0.001) and the absence of bacterial STIs among cSW (interaction HR = 0.34, *p* = 0.032) with discontinuation were also attenuated. No other significant interactions were identified, suggesting that the remaining associations were broadly consistent across key populations.

**FIGURE 3:**
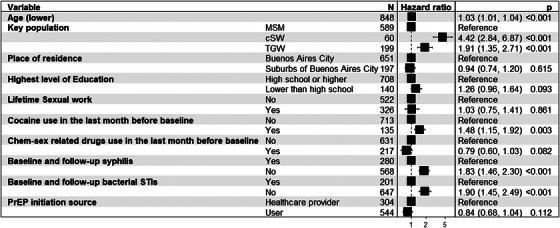
Cox regression model including demographic and behavioural factors. Adjusted hazard ratios for factors associated with time to first PrEP discontinuation. Squares indicate point estimates and horizontal lines represent 95% confidence intervals. *p*‐values *<* 0.05 indicate statistically significant factors associated with first PrEP discontinuation. cSW stands for cisgender sex workers, MSM stands for men who have sex with men, TGW stands for transgender women.

In the multivariable model (Figure 3), age, key population group, cocaine use in the last month before baseline and syphilis or other bacterial STIs (baseline or follow‐up) remained significantly associated with PrEP discontinuation. Younger age increased discontinuation (HR = 1.03, 95% CI: 1.01−1.04; *p* < 0.001). Compared with MSM, cSW and TGW had higher risk of discontinuation (cSW HR = 4.42, 95% CI: 2.84–6.87; *p* < 0.001; TGW HR = 1.91, 95% CI: 1.35–2.71; *p* < 0.001). Cocaine use in the last month before baseline was also associated with increased risk (HR = 1.48, 95% CI: 1.15–1.92; *p*: 0.003), while absence of baseline or follow‐up syphilis (HR = 1.83, 95% CI: 1.46−2.30; *p* < 0.001) and absence of other bacterial STIs at baseline or during follow‐up (HR = 1.90, 95% CI: 1.45−2.49; *p* < 0.001) were also associated with an increased risk of discontinuation. After adjustment, lifetime sex work (*p*: 0.861), chemsex‐associated drug use (*p*: 0.082) and provider‐initiated PrEP (*p*: 0.11) were no longer statistically significant.

### 3.4 Time and Categories for Discontinuation

**FIGURE 4:**
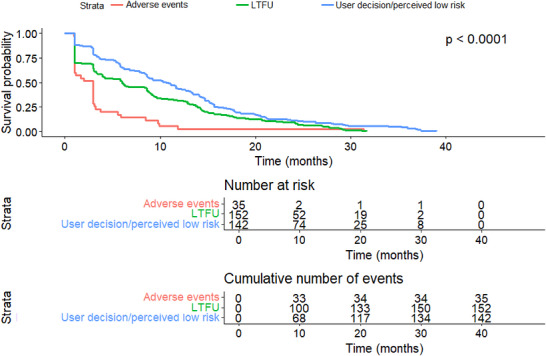
Kaplan−Meier curves of PrEP persistence by discontinuation category. Kaplan–Meier survival estimates show the probability of remaining on PrEP over time among those who ended up discontinuing PrEP due to adverse events, LTFU (lost to follow‐up) and user decision/perceived low risk. Persistence was significantly different across groups (log‐rank test, *p* < 0.0001). The lower panels display the number of participants at risk and the cumulative number of discontinuation events at each time point. Time is shown in months.

TFD varied significantly by discontinuation category. At 6 months, discontinuation probability was 86% among those reporting AEs, 49% among LTFU and 35% among those who discontinued by user decision/perceived low risk of HIV acquisition. At 12 months, these increased to 97%, 67% and 56%, respectively (Figure 4). Restricting the analysis to 12 months, mean time on PrEP was 4.95 months shorter for participants who discontinued due to AEs compared with those discontinuing by user decision/perceived low risk (95% CI: –6.21 to –3.62; *p <*0.001) and 3.11 months shorter compared with those LTFU (95% CI: –4.40 to –1.82; *p <*0.001). Participants LTFU had a mean time on PrEP 1.84 months shorter than those discontinued due to user decision/perceived low risk (95% CI: –2.85 to –0.82; *p* < 0.001).

Sociodemographic and behavioural profiles differed by discontinuation categories. Participants discontinuing due to user decision/perceived low risk were mostly MSM (62%; *p* = 0.003), whereas TGW and cSW were more frequent among those discontinuing due to AEs (46% and 26%, respectively). Participants LTFU had lower education (67% completed high‐school), than those discontinuing due to user decision/perceived low risk (86% completed high‐school; *p* < 0.001). Cocaine use prior to discontinuation was significantly more frequent among those discontinuing due to AEs (29%) or LTFU (28%) than those discontinuing by user decision/perceived low risk (7%; *p <*0.001). In a sensitivity analysis, censoring participants classified as LTFU at their last recorded visit yielded results consistent with the main findings.

## 4 Discussion

This study provides one of the first real‐world assessments of PrEP persistence within Argentina's national programme. Persistence was slightly above 50% in a cohort of key populations followed for up to 3 years. This level of discontinuation may limit the long‐term impact of oral PrEP within comprehensive HIV prevention strategies. Persistence declined steadily over time, and the main discontinuation categories were LTFU and user decision/perceived low HIV risk, while AEs accounted for a smaller proportion. HIV seroconversion was uncommon, accounting for 4% of discontinuations.

Our findings are consistent with PrEP implementation data from Latin America, including ImPrEP and PrEParadas, which suggest that persistence is shaped more by behavioural and structural factors than by medication safety [21, 22, 23, 24, 25]. In this context, the role of PrEP in Latin America increasingly depends on delivering acceptable and sustainable prevention to populations bearing the greatest HIV burden. Emerging modalities may help address barriers such as daily pill burden and oral tolerability, and studies from Brazil and Argentina suggest substantial interest in long‐acting injectable PrEP [26, 27, 28]. However, barriers such as perceived low HIV risk, disengagement from care, and structural vulnerability are likely to persist across modalities and will continue to require targeted programming strategies.

Within our cohort, TGW and cSW showed higher risks of discontinuation than MSM, consistent with prior evidence on structural vulnerability, including discrimination in healthcare settings, limited gender‐affirming services, economic insecurity, violence and substance use [14, 22, 29]. Still, effect modification by key population was limited, suggesting that most factors associated with discontinuation operated similarly across groups. Younger age, lower educational attainment and cocaine use were associated with lower persistence, whereas syphilis and other bacterial STIs were associated with higher persistence, supporting prevention‐effective interpretations in which PrEP use aligns with periods of higher HIV exposure [21, 23, 30, 31, 32, 33, 34]. Time to discontinuation varied substantially by categories. AEs were linked to earlier discontinuation, whereas LTFU and perceived low risk showed more gradual attrition, indicating that different pathways out of PrEP require different responses. Together, these findings support differentiated PrEP delivery within a combination prevention framework, particularly for TGW and cSW, with stronger follow‐up during the first months after initiation, counselling on real versus perceived risk, clear re‐engagement pathways and integrated models that address socioeconomic vulnerability, gender‐affirming care, violence and substance use [35].

This study has several limitations. The retrospective design may limit causal inference and introduces the possibility of misclassification arising from incomplete or imperfect routinely collected clinical data, particularly regarding categories and timing for discontinuation. Because most participants used daily PrEP, discontinuation was estimated based on daily pill coverage; however, among the small proportion using event‐driven PrEP, some participants classified as having discontinued may actually have remained on PrEP, potentially overestimating discontinuation. Although self‐reported adherence was collected through a structured questionnaire, it was not considered reliable for analysis because missed doses may have been confused with delayed doses. LTFU was included as an operational discontinuation category because it reflected interruption in PrEP dispensing and disengagement from follow‐up, but it may also have included unobserved PrEP use elsewhere. Because this study evaluated the first discontinuation, individuals who later restarted PrEP were not accounted for; if re‐initiations had been incorporated, the apparent impact of discontinuations would likely be smaller. STI diagnoses were likely underestimated due to limited access to diagnostic testing. Behavioural data were derived from a non‐validated self‐administered questionnaire, which may be subject to recall and social desirability bias. Qualitative insights on PrEP perceptions, motivations and barriers were not available. These limitations underscore the need for prospective and mixed‐method studies to better understand PrEP engagement in Argentina. Nonetheless, strengths include the large sample size, long follow‐up, inclusion of diverse key population groups and use of standardized data collected for the national PrEP programme.

## 5 Conclusions

Nearly half of the cohort discontinued PrEP over 3 years, with differences across key populations. Younger users, TGW and cSW had higher discontinuation risk, driven by social and structural constraints rather than clinical factors. STIs were associated with higher persistence, consistent with prevention‐effective adherence frameworks. These findings underscore the need for differentiated, community‐targeted and gender‐affirming PrEP delivery models that address structural vulnerability and harm‐reduction needs to improve PrEP engagement among key populations in Argentina and across Latin America. They also inform implementation of emerging PrEP modalities, as long‐acting options may reduce some barriers to persistence, while others still require tailored retention and counselling strategies.

## Author Contributions

JLG, DS, CC and MIF conceived the study and led its design and implementation. All authors participated in drafting the protocol and operational procedures. CC and MIF supervised data collection and clinical procedures. GM conducted the data analysis. JLG and CC drafted the manuscript. All authors read, reviewed and approved the final manuscript.

## Funding

This research received no specific grant from any funding agency in the public, commercial or not‐for‐profit sectors.

## Conflicts of Interest

The authors declare that they have no conflicts of interest.

## Data Availability

The study was conducted in accordance with the principles of the Declaration of Helsinki and was approved by the Institutional Review Board of Fundación Huésped (Buenos Aires, Argentina). The analysis was based on anonymized data, and an irreversible dissociation process was implemented to ensure confidentiality.
